# IVF/ICSI treatment for patients with diminished ovarian reserve with or without Kuntai capsule pretreatment: a retrospective cohort study stratified by a controlled ovarian stimulation regimen

**DOI:** 10.3389/fendo.2025.1598998

**Published:** 2025-06-05

**Authors:** Xiaoju Wan, Min Yu, Xingwu Wu, Zhihui Huang, Jun Tan

**Affiliations:** Reproductive Medicine Center, Jiangxi Maternal and Child Health Hospital, Nanchang, China

**Keywords:** diminished ovarian reserve, Kuntai capsule, infertility, clinical pregnancy, antagonists

## Abstract

**Background:**

Kuntai capsules, a traditional Chinese medicine, are speculated to improve the treatment outcomes of patients with ovarian reserve dysfunction (DOR), but existing evidence is limited.

**Objective:**

To investigate the effects of Kuntai capsule pretreatment on the IVF/ICSI treatment outcomes of DOR patients with different ovarian stimulation regimens (PPOS, antagonists, and microstimulation).

**Method:**

A retrospective cohort study design was used to include 7271 DOR patients who underwent IVF/ICSI between January 2015 and February 2025. After baseline data were balanced through propensity score matching (PSM), 1474 patients were ultimately included. The number of retrieved eggs, laboratory indicators, and clinical outcomes were compared between the group pretreated with Kuntai capsules and the group not pretreated with Kuntai capsules under three ovarian stimulation regimens, and confounding factors were controlled via a generalized estimating equation (GEE) model.

**Result:**

In the PPOS regimen, the number of retrieved eggs (without kuntai: 2.00 [1.00;4.00], with kuntai: 2.00 [1.00;3.00], p<0.001) and normal fertilized eggs (without kuntai:2.00 [1.00;3.00], with kuntai: 2.00 [1.00;2.00], p=0.004) in the Kuntai pretreatment group significantly decreased, but the embryo utilization rate increased (without kuntai:101 (69.2%), with kuntai: 79 (74.5%), p=0.012). There was no difference between the two groups in the antagonist regimen. The Kuntai group had a higher failure rate for egg retrieval in the microstimulation program (without kuntai: 0 (0.00%), with kuntai:6 (6.25%), p=0.029). Among the three regimens, Kuntai pretreatment did not significantly improve the clinical pregnancy rate, live birth rate, or other outcomes (all p>0.05). Age stratification analysis and GEE analysis did not reveal significant differences.

**Conclusion:**

Pretreatment with Kuntai capsules did not significantly improve the number of retrieved eggs or clinical pregnancy outcomes in DOR patients under different ovarian stimulation regimens, and its application effect is limited. Further verification through prospective research is needed in the future.

## Introduction

With the increasing number of women who marry and have children late, the demand for childbirth among elderly women is increasing. Diminished ovarian reserve (DOR) is a physiological phenomenon associated with a decrease in the number and quality of ovarian follicles in women as they age and is characterized by decreased fertility, menstrual irregularities, and fluctuations in sex hormones (1). Although *in vitro* fertilization and embryo transfer techniques have become the main treatment methods for DOR infertility, DOR patients still face problems such as insufficient egg retrieval, low rates of high-quality embryos, and low clinical pregnancy rates, which are difficult issues in the field of reproduction (2, 3).

In clinical practice, an appropriate controlled ovarian stimulation (COS) regimen is an important strategy to improve the outcomes of DOR patients. However, for DOR patients, there is still no conclusion on how to choose an appropriate COS regimen. Compared with traditional agonist regimens, gonadotropin-releasing hormone (GnRH) antagonists, microsimulation, and progesterone-induced ovarian stimulation (PPOS) regimens are simple, have shorter cycles, and are commonly used in DOR patients (4–8).

The Kuntai capsule is the first traditional Chinese patent medicine and simple preparation approved in China to treat diseases related to ovarian function decline, and its formula comes from the Treatise on Febrile Diseases and Miscellaneous Diseases (9). According to Traditional Chinese Medicine (TCM), the pathological mechanism of DOR involves “deficiency of the spleen and kidney, insufficiency of Tian Gui (heavenly endowment), and deficiency of the Chong and Ren meridians.” The TCM treatment approach focuses on tonifying the kidney, nourishing the liver, fortifying the spleen, boosting qi, enriching blood, and promoting meridian circulation (9). Kun Tai Capsule, composed of Rehmannia glutinosa (prepared rehmannia root), Coptis chinensis (coptis root), Paeonia lactiflora (white peony root), donkey-hide gelatin (Ejiao), Scutellaria baicalensis (baical skullcap root), and Poria cocos (poria fungus), serves as a formula to nourish the kidney, enrich yin, clear heat, and calm the mind. Multiple studies have indicated that Kuntai capsules can be used for the treatment of clinical diseases such as menopausal syndrome and ovarian reserve dysfunction (10–12). In *in vitro* fertilization (IVF) or intracytoplasmic sperm injection (ICSI) cycles, some studies have shown that Kuntai capsules can effectively improve the ovarian response and embryo quality in DOR patients (11, 13). However, the existing research sample sizes are mostly less than 200 cases and are often combined with estradiol, acupuncture, moxibustion and other means, which limits the strength of the evidence and the promotion of conclusions.

The speculation of this study is that pretreatment with Kuntai capsules can improve the outcome of IVF/ICSI treatment in DOR patients and that the improvement effect is related to the ovulation stimulation regimen. Different ovulation stimulation programs have different improvement effects. Therefore, we designed a large-sample retrospective cohort study stratified by the COS regimen to provide doctors with a quantitative reference standard for the use of Kuntai capsules.

## Materials and methods

### Study design and population

From January 2015 to February 2025, 7271 DOR patients who underwent IVF or ICSI and used antagonist, microsimulation, or PPOS COS regimens at the Jiangxi Maternal and Child Health Hospital, Reproductive Medicine Center, were retrospectively identified. The Reproductive Medicine Ethics Committee of Jiangxi Maternal and Child Health Hospital approved this study (SZYY-202504).

The inclusion criteria were as follows: ① infertility duration ≥ 1 year; ② anti-Müllerian hormone (AMH) < 1.1 ng/ml, antral follicle count (AFC) < 7, or basal follicle stimulating hormone (FSH) ≥ 10 IU/L; and ③ the COS regimen included antagonists, PPOS, or microsimulation. The exclusion criteria were as follows: ① chromosomal abnormalities; ② congenital uterine malformation or organic uterine lesions; ③ severe hydrosalpinx; ④ history of recurrent miscarriage; ⑤ egg and sperm supply cycle; ⑥ outcome measures lost to follow-up; and ⑦ egg retrieval cancelled due to follicular dysplasia. A total of 7271 infertile couples with fresh IVF/ICSI were included in this study, including 737 cases with Kuntai capsule pretreatment and 6534 cases without Kuntai capsule pretreatment. In the Kuntai capsule pretreatment group, 596 patients received the PPOS regimen, 45 patients received the antagonist regimen, and 96 patients received the microsimulation regimen. In the without Kuntai capsule pretreatment group, 2445 patients received the PPOS regimen, 2512 patients received the antagonist regimen, and 1577 patients received the microsimulation regimen.

**Figure 1:**
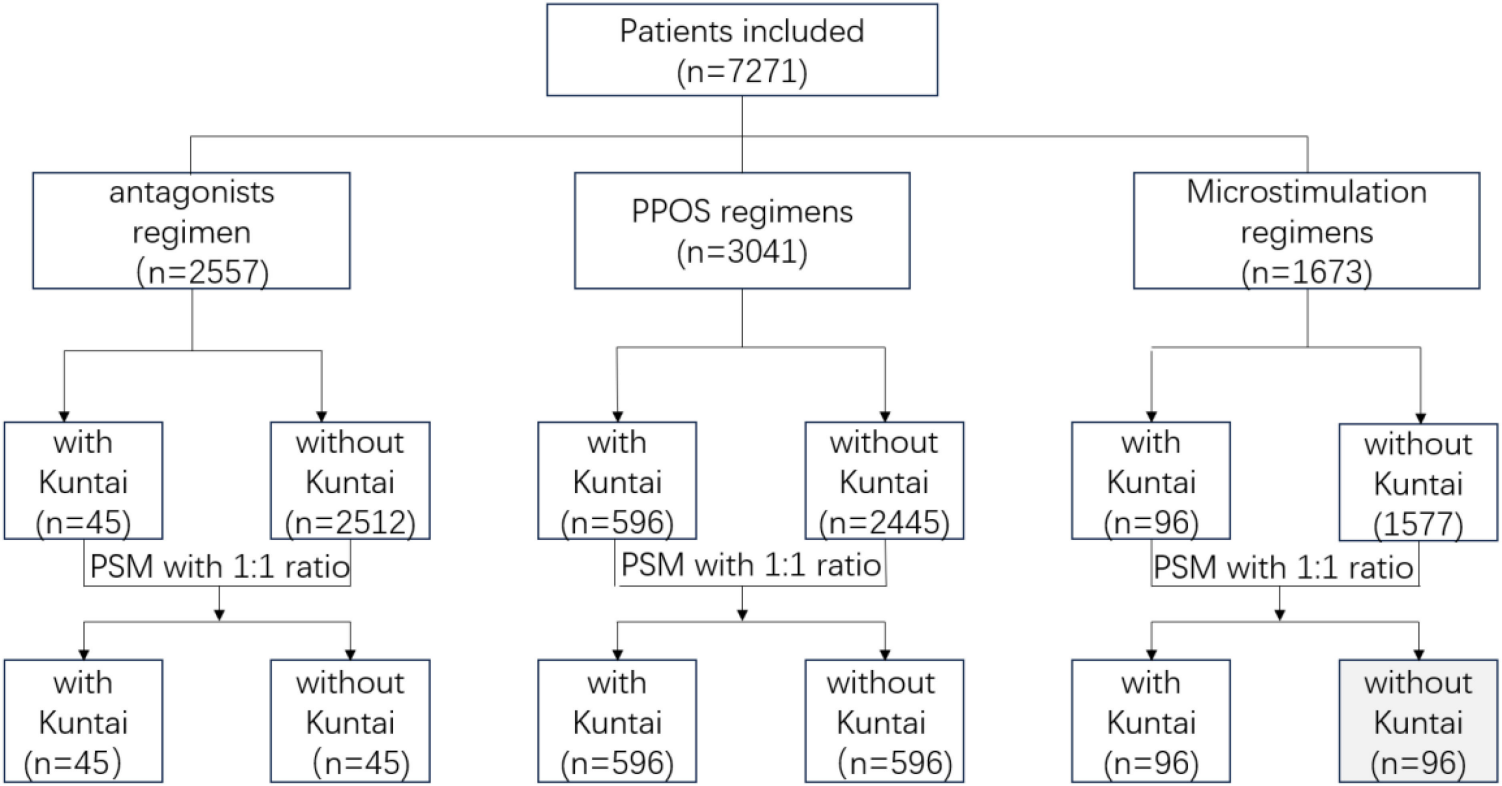
Flow chart of the study.

Considering that the sample size ratio between the groups exceeded the range of 1:4, the propensity score matching (PSM) method was used to balance the baseline data of each group, including female age, body mass index (BMI), ovarian reserve (basal FSH, AFC, AMH), infertility type, infertility duration, and infertility factors, which were matched at a 1:1 ratio. Finally, 1474 patients were included in the study, and the research process is illustrated in **Figure 1**.

### COS regimen and pretreatment of Kuntai capsules

PPOS regimen: Starting from the 2nd to 3rd day of the menstrual cycle, oral medroxyprogesterone acetate tablets (medroxyprogesterone acetate; Zhejiang Xianju) at a dosage of 8–10 mg/d will be taken daily, while injectable urinary follicle stimulating hormone (Lishenbao, Shanghai Lizhu) at a dosage of 150–300 U/d will be administered until the trigger day.

Antagonist regimen: Starting from the 2nd to 3rd day of the menstrual cycle, human menopausal gonadotropin (HMG, Le Baode; Zhuhai Lizhu) (150~300 U) was injected intramuscularly every day. When one dominant follicle exceeds 13 mm or estrogen exceeds 600 pg/ml, 0.25 mg of Ganirelix acetate (Merck, USA) was subcutaneously injected once a day until the trigger day.

Microstimulation regimen: Starting from the 2nd to 3rd day of the menstrual cycle, clomifene citrate tablets (Fadiland; Gaote, Cyprus) 100 mg or letrozole (Furui, Jiangsu Hengrui) 2.5 mg once a day, along with intramuscular injection of human menopausal gonadotropin (HMG, Le Baode; Zhuhai Lizhu) 150 U/d until the trigger day, are taken.

Pretreatment of Kuntai capsules: Kuntai capsules (Guiyang Xintian, 0.5 g/capsule) were taken orally on the third day of menstruation, with 4 capsules each time, 3 times a day, and continued until the day of egg retrieval.

### Egg retrieval, embryo culture and fresh embryo transfer

When there is one follicle with a diameter ≥ 18 mm or three follicles with a diameter ≥ 17 mm, a subcutaneous injection of 0.2 mg of triptorelin acetate (Dabijia, Germany) and an intramuscular injection of human chorionic gonadotropin (hCG, Shanghai Lizhu) 2000 U are given to induce ovulation. After 34–36 h, eggs are collected via transvaginal ultrasound puncture.

IVF/ICSI fertilization was performed after egg retrieval. The embryos were cultured sequentially in G1/G2 (Vitrlife, Sweden), and on the third day, the quality of the cleavage-stage embryos was evaluated according to the Cummins criteria. Partial embryos were further cultured until days 5/6, and blastocyst grading was performed according to Gardner’s criteria.

After ovulation induction with the antagonist regimen and the letrozole microstimulation regimen, the endometrial condition and serum hormone levels were evaluated. If there are no contraindications for transplantation, 1–2 cleavage-stage embryos or blastocysts should be selected for fresh embryo transfer. After the PPOS regimen and the clomifene citrate tablet microstimulation regimen, whole embryo freezing was performed, and thawed embryo transfer was performed at a later date.

### Endometrial preparation and frozen-thawed embryo transfer

For frozen-thawed embryo transfer (FET), natural cycles, artificial cycles, or regulated artificial cycles are used for endometrial preparation.

The natural cycle is suitable for individuals with normal ovulation. Follicle size and sex hormone levels were monitored on the 12th day of menstruation. Inject 40–60 mg/d progesterone (Zhejiang Xianju) intramuscularly after the luteinizing hormone (LH) peak.

Artificial cycle: This cycle is used mainly for people with infrequent or irregular menstruation. Starting from the 2nd to 3rd day of menstruation, oral estradiol valerate (Bujiale, Bayer, Germany) at a dosage of 4–8 mg/d was administered. After 12–14 days, vaginal ultrasound confirmed that no dominant follicle developed, and progesterone (Zhejiang Xianju) at a dosage of 80 mg/d was administered to transform the endometrium.

Downregulated artificial cycles: These cycles are suitable mainly for patients with a history of previous intrauterine adhesion surgery or cesarean section. A subcutaneous injection of 3.75 mg of leuprorelin acetate (Beiyi, Shanghai Lizhu) was administered from the 2nd to 3rd day of menstruation, and the medication regimen was the same as the artificial cycle after 28 days of adjustment.

The ovulation induction cycle is suitable for patients with natural cycle follicular dysplasia or those who wish to shorten the preparation time. On the 2nd to 3rd days of menstruation, oral letrozole (Furui, Jiangsu Hengrui) 2 (5 mg/time) was administered once a day for 5 consecutive days. On the 10th to 12th days of menstruation, ultrasound examination was performed, and urinary follicle stimulating hormone (HMG, Le Baode; Zhuhai Lizhu) was added once a day according to the development of follicles, 150 U via intramuscular injection. When the thickness of the endometrium is ≥ 8 mm, urinary luteinizing hormone (LH) testing is performed. When LH ≤ 20 mIU/ml, human chorionic gonadotropin (hCG, Shanghai Lizhu) 4000 IU is injected intramuscularly. After ovulation was detected by ultrasound, oral administration of 10 mg/dose progesterone was started 3 times a day, and the vaginal use of progesterone soft capsules was 200 mg/dose 2 times a day.

One to two cleavage-stage embryos were transferred on the 4th day after the endometrium was converted, or 1–2 blastocysts were transferred on the 6th day.

### Luteal support and pregnancy observation

Fresh embryo transfer: Starting from the day of egg retrieval, 40 mg of progesterone (Xianju, Zhejiang) was intramuscularly injected twice a day until the day of transplantation. Starting from the day after transplantation, progesterone capsules (Angel Tan; Besins Iscovsco, France) were administered vaginally at a dose of 0.2 g 3 times a day, and progesterone capsules (Yimaxin; Zhejiang Xianju) were taken orally at a dose of 100 mg 2 times a day until 10 weeks of pregnancy.

Frozen-thawed embryo transfer (FET): From the date of transplantation, 20 mg/d oral dydrogesterone (Duffton, Abbott, Netherlands) combined with 90 mg/d vaginal progesterone sustained-release gel (Seroton, Merck Seranol, Germany) was given, and the luteal support lasted until 10 weeks of pregnancy.

Fourteen days after embryo transfer, a positive HCG blood test (HCG≥5 mIU/ml) indicated a biochemical pregnancy. Five weeks after transplantation, B-ultrasound examination revealed a gestational sac and primitive pulsation in the uterine cavity, which confirmed clinical pregnancy.

### Outcome measures

The main observation indicator was the number of retrieved eggs. The definition of secondary observation indicators is as follows:

Oocyte failure rate= Number of cycles with 0 retrieved eggs/number of egg retrieval cycles in the group;
MII oocyte rate = Number of MII oocytes/Total retrieved oocytes;
Normal fertilization rate=number of double prokaryotic fertilized eggs/total number of retrieved eggs × 100%;
Normal cleavage rate=number of cleavage-stage embryos/number of normal fertilized eggs × 100%;
D3 high-quality embryo rate=number of D3 high-quality embryos/number of cleavage-stage embryos × 100%;
Available embryo formation rate = number of available embryos/number of cleavage-stage embryos ×100%;
The rate of unusable embryos=the number of unusable embryo cycles in a group/the total number of egg retrieval cycles in that group ×100%;
Biochemical pregnancy rate= number of biochemical clinical cycles/number of transplant cycles × 100%;
Planting rate=number of gestational sacs/number of transplanted embryos × 100%;
Clinical pregnancy rate=number of clinical pregnancy cycles/number of transplant cycles × 100%;
Pregnancy loss rate = number of natural or therapeutic abortion cycles/number of clinical pregnancy cycles × 100%;
The live birth rate was calculated as the number of live births at ≥ 28 weeks of pregnancy/number of transplant cycles × 100%.

### Data statistics and analysis

R 3.3.4 was used for statistical analysis of the data. The Shapiro–Wilk test was used to test the normality of the measurement data. Normally distributed data are presented as the means eastandard deviations ( $\bar{X}\pm S$ ), and intergroup comparisons were conducted via t-tests. Quantitative data that conform to a skewed distribution or approximate normal distribution are represented by medians [M (Q1, Q3)], and intergroup comparisons are performed via the Mann–Whitney U test. Count data are expressed as percentages (%) and were compared between groups via the chi square test or Fisher’s test. To control for confounding factors, the PSM method was used to balance two sets of baseline data. Considering the clustering of data (some patients contributed more than one cycle), the generalized estimation equation (GEE) based on a logistic regression model was used to control for the influence of confounding factors (14). The bilateral test method was used, and P<0.05 was considered statistically significant.

## Results

### Basic information of DOR patients in each group before and after matching

**Tables 1**–**3** display the baseline characteristics of the women before and after PSM for the PPOS regimen, antagonist regimen, and microstimulation regimen, respectively. Before PSM, there were significant differences (P < 0.05) in some baseline characteristics between the group without Kuntai pretreatment and the group with Kuntai pretreatment. After PSM, the baseline characteristics of women in both the group without Kuntai pretreatment and the group with Kuntai pretreatment were similar, and the number of patients was balanced.

**Table 1:** Baseline characteristics of patients with PPOS ovulation induction before and after PSM.

	Before matching			After matching		
	Without kuntai (n=2445)	With kuntai (n=596)	*p*	Without kuntai (n=596)	With kuntai (n=596)	*p*
Age(years)	37.0 [33.0, 40.0]	36.0 [32.0, 40.0]	0.202	36.0 [32.0, 40.0]	36.0 [32.0, 40.0]	0.755
BMI (kg/m^2^)	22.0 [20.2, 24.0]	22.0 [20.3, 24.0]	0.810	22.0 [20.2, 24.0]	22.0 [20.3, 24.0]	0.881
Basic FSH (IU/L)	8.21 [6.41, 11.1]	9.06 [6.83, 12.3]	<0.001	8.99 [6.82, 12.1]	9.06 [6.83, 12.3]	0.697
Basic E_2_ (pg/ml)	35.2 [25.3, 50.3]	34.1 [24.1, 51.2]	0.391	34.7 [25.0, 50.3]	34.1 [24.1, 51.2]	0.816
Basic LH (IU/L)	3.72 [2.60, 5.29]	4.28 [3.00, 5.82]	<0.001	4.02 [2.89, 6.04]	4.28 [3.00, 5.82]	0.617
AMH (ug/L)	0.63 [0.39, 0.91]	0.54 [0.29, 0.81]	<0.001	0.56 [0.33, 0.79]	0.54 [0.29, 0.81]	0.730
AFC	5.00 [3.00, 6.00]	5.00 [3.00, 5.00]	0.411	5.00 [3.00, 5.00]	5.00 [3.00, 5.00]	0.425
Infertility duration (years)	3.00 [2.00, 6.00]	3.00 [2.00, 6.00]	0.201	3.00 [2.00, 6.00]	3.00 [2.00, 6.00]	0.653
Infertility type, n (%)			0.497			0.708
Second	1699 (69.5%)	405 (68.0%)		412 (69.1%)	405 (68.0%)	
Primary	746 (30.5%)	191 (32.0%)		184 (30.9%)	191 (32.0%)	
Infertility factors, n (%)			0.053			0.622
Tubal factor	827 (33.8%)	181 (30.4%)		166 (27.9%)	181 (30.4%)	
Male factor	655 (26.8%)	148 (24.8%)		156 (26.2%)	148 (24.8%)	
other	963 (39.4%)	267 (44.8%)		274 (46.0%)	267 (44.8%)	

Numerical variables that follow a normal distribution are reported as mean (standard deviation), numerical variables that do not follow a normal distribution are reported as median [interquartile range], and categorical variables are reported as numbers (percentage). Continuous variables are analyzed using the Mann-Whitney U test, while categorical variables are analyzed using the chi-square test or Fisher’s test.

Other infertility factors include ovulation disorders, endometriosis, uterine factors, and so on.

BMI, body mass index; FSH, follicle stimulating hormone; E_2_, estradiol; LH, luteinizing hormone; AMH, anti-Müllerian hormone; AFC, antral follicle count.

**Table 2:** Baseline characteristics of patients with antagonist ovulation induction before and after PSM.

	Before matching			After matching		
	Without kuntai (n=2512)	With kuntai (n=45)	*p*	Without kuntai (n=45)	With kuntai (n=45)	*p*
Age(years)	36.0 [32.0, 40.0]	37.0 [32.0, 40.0]	0.600	37.1 (5.17)	36.2 (4.83)	0.402
BMI (kg/m^2^)	21.9 [20.1, 24.2]	22.8 [19.6, 24.5]	0.668	23.1 (3.11)	22.5 (3.27)	0.375
Basic FSH (IU/L)	7.89 [6.28, 10.2]	8.30 [6.78, 9.09]	0.907	7.30 [5.64, 8.83]	8.30 [6.78, 9.09]	0.302
Basic E_2_ (pg/ml)	35.5 [25.6, 49.0]	37.1 [26.6, 49.2]	0.717	34.4 (15.6)	38.4 (14.2)	0.213
Basic LH (IU/L)	3.70 [2.62, 5.15]	3.67 [2.65, 4.82]	0.988	3.18 [2.46, 3.99]	3.67 [2.65, 4.82]	0.130
AMH (ug/L)	0.75 [0.52, 1.07]	0.66 [0.54, 0.89]	0.111	0.75 [0.55, 0.97]	0.66 [0.54, 0.89]	0.410
AFC	5.00 [4.00, 6.00]	5.00 [4.00, 5.00]	0.411	5.00 [3.00, 6.00]	5.00 [4.00, 5.00]	0.615
Infertility duration (years)	3.00 [2.00, 6.00]	3.00 [2.00, 6.00]	0.852	4.00 [2.00, 8.00]	3.00 [2.00, 6.00]	0.158
Infertility type, n (%)			0.786			0.806
Second	1767 (70.3%)	33 (73.3%)		35 (77.8%)	33 (73.3%)	
Primary	745 (29.7%)	12 (26.7%)		10 (22.2%)	12 (26.7%)	
Infertility factors, n (%)			0.687			0.967
Tubal factor	973 (38.7%)	16 (35.6%)		15 (33.3%)	16 (35.6%)	
Male factor	639 (25.4%)	14 (31.1%)		15 (33.3%)	14 (31.1%)	
other	900 (35.8%)	15 (33.3%)		15 (33.3%)	15 (33.3%)	

Numerical variables that follow a normal distribution are reported as mean (standard deviation), numerical variables that do not follow a normal distribution are reported as median [interquartile range], and categorical variables are reported as numbers (percentage).

Continuous variables are analyzed using the Mann-Whitney U test, while categorical variables are analyzed using the chi-square test or Fisher’s test.

Other infertility factors include ovulation disorders, endometriosis, uterine factors, and so on.

BMI, body mass index; FSH, follicle stimulating hormone; E_2_, estradiol; LH, luteinizing hormone; AMH, anti-Müllerian hormone; AFC, antral follicle count.

**Table 3:** Baseline characteristics of patients with Micro stimulation ovulation induction before and after PSM.

	Before matching			After matching		
	Without kuntai (n=1577)	With kuntai (n=96)	*p*	Without kuntai (n=96)	With kuntai (n=96)	*p*
Age(years)	38.0 [33.0, 41.0]	37.0 [33.0, 40.0]	0.115	37.0 [32.0, 40.0]	37.0 [33.0, 40.0]	0.948
BMI (kg/m^2^)	22.0 [20.3, 24.0]	22.6 [20.3, 24.4]	0.369	22.4 (2.64)	22.6 (2.97)	0.599
Basic FSH (IU/L)	8.77 [6.80, 12.0]	9.39 [7.39, 12.3]	0.203	8.45 [7.06, 11.6]	9.39 [7.39, 12.3]	0.285
Basic E_2_ (pg/ml)	35.4 [24.5, 48.3]	35.2 [19.1, 47.9]	0.394	32.1 [23.6, 48.2]	35.2 [19.1, 47.9]	0.822
Basic LH (IU/L)	3.74 [2.70, 5.23]	3.80 [2.58, 5.52]	0.942	3.70 [2.80, 5.76]	3.80 [2.58, 5.52]	0.677
AMH (ug/L)	0.66 [0.39, 0.76]	0.57 [0.37, 0.85]	0.423	0.66 [0.47, 0.89]	0.57 [0.37, 0.85]	0.072
AFC	4.00 [3.00, 5.00]	4.50 [3.00, 5.25]	0.417	5.00 [4.00, 6.00]	4.50 [3.00, 5.25]	0.134
Infertility duration (years)	4.00 [2.00, 7.00]	3.00 [2.00, 6.00]	0.951	3.00 [2.00, 7.00]	3.00 [2.00, 6.00]	0.692
Infertility type, n (%)			0.367			0.764
Second	1112 (70.5%)	63 (65.6%)		60 (62.5%)	63 (65.6%)	
Primary	465 (29.5%)	33 (34.4%)		36 (37.5%)	33 (34.4%)	
Infertility factors, n (%)			0.649			0.768
Tubal factor	646 (41.0%)	35 (36.5%)		35 (36.5%)	35 (36.5%)	
Male factor	416 (26.4%)	26 (27.1%)		30 (31.2%)	26 (27.1%)	
other	515 (32.7%)	35 (36.5%)		31 (32.3%)	35 (36.5%)	

Numerical variables that follow a normal distribution are reported as mean (standard deviation), numerical variables that do not follow a normal distribution are reported as median [interquartile range], and categorical variables are reported as numbers (percentage).

Continuous variables are analyzed using the Mann-Whitney U test, while categorical variables are analyzed using the chi-square test or Fisher’s test.

Other infertility factors include ovulation disorders, endometriosis, uterine factors, and so on.

BMI, body mass index; FSH, follicle stimulating hormone; E_2_, estradiol; LH, luteinizing hormone; AMH, anti-Müllerian hormone; AFC, antral follicle count.

### Ovulation induction outcomes and laboratory outcomes of DOR patients in each group after PSM

In patients receiving the PPOS regimen, compared with those who did not use Kuntai, the group that used Kuntai had lower estradiol (E_2_) values on the trigger day (without kuntai: 556 [354;821], with kuntai:510 [334;731], p=0.034), fewer retrieved eggs (without kuntai: 2.00 [1.00;4.00], with kuntai:2.00 [1.00;3.00], p<0.001) but no difference in the MII oocyte rate, a lower number of normal fertilized eggs (without kuntai:2.00 [1.00;3.00], with kuntai:2.00 [1.00;2.00], p=0.004), a lower number of normal fertilized cleavages (without kuntai:2.00 [1.00;3.00], with kuntai:2.00;2.00], p=0.009), and a higher utilization rate of embryos (without kuntai:101 (69.2%), with kuntai:79 (74.5%), p=0.012) (**Table 4**).

**Table 4:** Ovulation induction outcomes and laboratory outcomes of DOR patients in each group after PSM.

	PPOS			Antagonist			Micro stimulation		
	Without kuntai (n=596)	With kuntai (n=596)	*p*	Without kuntai (n=45)	With kuntai (n=45)	*p*	Without kuntai (n=96)	With kuntai (n=96)	*p*
Total dose of Gn (U)	1575 [1200;2025]	1500 [1125;2025]	0.192	2100 [1800;2400]	2300 [1800;2700]	0.144	1050 [600;1425]	900 [600;1200]	0.135
Dosing days of Gn (day)	8.00 [7.00;10.0]	9.00 [7.00;10.0]	0.125	8.00 [7.00;10.0]	8.00 [8.00;10.0]	0.844	6.50 [4.00;9.00]	6.00 [4.00;8.00]	0.212
E_2_ on trigger day (pg/mL)	556 [354;821]	510 [334;731]	0.034	734 [482;1021]	753 [511;1364]	0.302	212 [117;524]	156 [94.2;371]	0.032
LH on trigger day (IU/L)	3.13 [1.96;5.06]	3.30 [2.14;4.78]	0.389	2.48 [1.81;4.15]	3.31 [2.07;5.51]	0.160	4.04 [2.44;6.69]	4.54 [2.58;6.78]	0.500
P on trigger day (ng/mL)	0.20 [0.11;0.31]	0.19 [0.12;0.30]	0.379	0.35 [0.20;0.59]	0.41 [0.19;0.65]	0.526	0.30 [0.24;0.55]	0.30 [0.20;0.39]	0.038
Oocyte not obtained rate, n (%)	25 (4.19%)	30 (5.03%)	0.581	1 (2.22%)	0 (0.00%)	1.000	0 (0.00%)	6 (6.25%)	0.029
Number of oocytes retrieved	2.00 [1.00;4.00]	2.00 [1.00;3.00]	<0.001	4.00 [2.00;6.00]	4.00 [3.00;5.00]	0.977	2.00 [1.00;3.00]	2.00 [1.00;3.00]	0.247
MII oocyte rate, n (%)	1430 (85.1%)	1227(85.4%)	0.874	144 (73.5%)	159 (80.7%)	0.112	143(70.8%)	184(75.4)	0.322
Fertilization^*^, n (%)			0.499			0.110			0.419
IVF	393 (69.6%)	376 (67.5%)		22 (50.0%)	31 (68.9%)		62 (64.6%)	62 (71.3%)	
ICSI or rescue icsi	172 (30.4%)	181 (32.5%)		22 (50.0%)	14 (31.1%)		34 (35.4%)	25 (28.7%)	
Number of normally fertilized embryos^*^	2.00 [1.00;3.00]	2.00 [1.00;2.00]	0.004	2.50 [1.00;4.00]	2.00 [2.00;4.00]	0.596	1.00 [1.00;2.00]	1.00 [1.00;2.00]	0.192
Number of cleavages in normally fertilized embryos^*^	2.00 [1.00;3.00]	2.00 [1.00;2.00]	0.009	2.00 [1.00;4.00]	2.00 [2.00;4.00]	0.599	1.00 [1.00;2.00]	1.00 [1.00;2.00]	0.083
Number of high-quality embryos on day 3^*^	1.00 [0.00;1.00]	0.00 [0.00;1.00]	0.353	1.00 [0.00;1.00]	1.00 [0.00;1.00]	0.647	0.00 [0.00;1.00]	0.00 [0.00;1.00]	0.828
number of available embryos^*^	1.00 [1.00;2.00]	1.00 [1.00;2.00]	0.190	2.00 [1.00;2.25]	2.00 [1.00;2.00]	0.701	1.00 [0.00;2.00]	1.00 [0.00;1.50]	0.349
Normal fertilization rate^*^, n (%)	150 (61.5%)	113 (57.4%)	0.343	122 (62.2%)	138 (70.1%)	0.126	150 (61.5%)	113 (57.4%)	0.437
Normal cleavage rate^*^, n (%)	146 (97.3%)	106 (93.8%)	0.200	118 (96.7%)	133 (96.4%)	1	146 (97.3%)	106 (93.8%)	0.215
high-quality embryos on day 3 rate^*^, n (%)	53 (36.3%)	41 (38.7%)	0.607	36 (30.5%)	46 (34.6%)	0.581	53 (36.3%)	41 (38.7%)	0.800
Available embryos formation rate^*^, n (%)	101 (69.2%)	79 (74.5%)	0.012	78 (66.1%)	84 (63.2%)	0.723	101 (69.2%)	79 (74.5%)	0.431
unusable embryos rate^*^, n (%)	29 (30.2%)	33 (37.9%)	0.980	7 (15.9%)	5 (11.1%)	0.725	29 (30.2%)	33 (37.9%)	0.344

Numerical variables that follow a normal distribution are reported as mean (standard deviation), numerical variables that do not follow a normal distribution are reported as median [interquartile range], and categorical variables are reported as numbers (percentage).

Continuous variables are analyzed using the Mann-Whitney U test, while categorical variables are analyzed using the chi-square test or Fisher’s test.

* Some patients were excluded from the statistical analysis of the variables marked with * due to egg freezing, poor egg quality resulting in no fertilization procedure, or failure to retrieve eggs. Therefore, for these variables marked with *, in the PPOS patients, the sample size (n) was 557 in the group with Kuntai and 565 in the group without Kuntai; in the antagonist patients, the sample size (n) was 45 in the group with Kuntai and 44 in the group without Kuntai; in the micro stimulation patients, the sample size (n) was 87 in the group with Kuntai and 96 in the group without Kuntai.

GN, gonadotropins; E2, estradiol; LH, luteinizing hormone; P, Progesterone; IVF, *in vitro* fertilization; ICSI, intracytoplasmic sperm injection.

In patients receiving the antagonist regimen, there was no difference in ovulation induction or laboratory outcomes between the group receiving Kuntai and the group not receiving Kuntai (**Table 4**).

In patients receiving the microstimulation regimen, compared with those not receiving Kuntai, the group receiving Kuntai had lower E_2_ values on the trigger day (without kuntai: 212 [117;524], with kuntai:156 [94.2;371], p=0.032), lower progesterone (P) values on the trigger day (without kuntai: 0.30 [0.24;0.55], with kuntai: 0.30 [0.20;0.39], p=0.038), and higher rates of oocyte failure (without kuntai: 0 (0.00%), with kuntai:6 (6.25%) , p=0.029) (**Table 4**).

### Clinical outcome analysis of thawing transplantation cycles

Among 1474 patients, 82 patients underwent fresh embryo transfer (82 fresh embryo transfer cycles), and 737 patients underwent frozen-thawed embryo transfer (FET) (26 patients had two FET cycles, for a total of 763 FET cycles). Considering the small number of fresh embryo transplant cycles, only the outcomes of FET cycles were analyzed.

Compared with the group not receiving Kuntai, the group receiving Kuntai had fewer transplanted embryos (without kuntai: 1.83 (0.44), with kuntai: 1.72 (0.48), p=0.004), but there was no difference in clinical outcomes, including the biochemical pregnancy rate, implantation rate, clinical pregnancy rate, pregnancy loss rate, and live birth rate.

In patients receiving both the antagonist regimen and the microstimulation regimen, there were no differences in clinical outcomes between the kuntai group and the without kuntai group (**Table 5**).

**Table 5:** Frozen-thawed embryo transfer clinical outcomes stratified by controlled ovarian stimulation regimen.

	PPOS group			Antagonist group			Micro stimulation group		
	Without kuntai (n=321^*^)	With kuntai (n=311^*^)	*p*	Without kuntai (n=19^*^)	With kuntai (n=15^*^)	*p*	Without kuntai (n=51^*^)	With kuntai (n=46^*^)	*p*
Endometrial preparation protocol, n (%)			0.344			1.000			0.882
Artificial cycle	94 (29.3%)	105 (33.8%)		4 (21.1%)	4 (26.7%)		19 (37.3%)	15 (32.6%)	
Downregulated artificial cycle	184 (57.3%)	157 (50.5%)		12 (63.2%)	10 (66.7%)		21 (41.2%)	19 (41.3%)	
Micro stimulation cycle	3 (0.93%)	5 (1.61%)		1 (5.26%)	0 (0.00%)		0 (0.00%)	1 (2.17%)	
Natural cycle	40 (12.5%)	44 (14.1%)		2 (10.5%)	1 (6.67%)		11 (21.6%)	11 (23.9%)	
Endometrial thickness (mm)	9.40 [8.30, 10.5]	9.30 [8.10, 10.4]	0.504	9.02 (1.96)	11.1 (2.31)	0.010	9.50 [8.45, 11.2]	9.50 [8.12, 10.5]	0.723
No. of embryos transferred	1.83 (0.44)	1.72 (0.48)	0.004	1.53 (0.51)	1.53 (0.52)	0.969	1.71 (0.50)	1.74 (0.44)	0.730
Stage of embryos transferred, n (%)			0.009			0.068			0.510
Cleavage	289 (90.0%)	257 (82.6%)		16 (84.2%)	8 (53.3%)	0.617	47 (92.2%)	40 (87.0%)	0.617
Blastocyst	32 (9.97%)	54 (17.4%)		3 (15.8%)	7 (46.7%)	0.730	4 (7.84%)	6 (13.0%)	0.730
Biochemical pregnancy rate, n (%)	186 (57.9%)	164 (52.7%)	0.216	8 (42.1%)	7 (46.7%)	1	17 (33.3%)	19 (41.3%)	0.548
Implantation rate, n (%)	174 (29.7%)	165 (30.8%)	0.724	7 (24.1%)	7 (30.4%)	0.846	20 (23.0%)	17 (21.3%)	0.933
Clinical pregnancy rate, n (%)	136 (42.5%)	141 (45.3%)	0.524	5 (26.3%)	6 (40.0%)	0.475	16 (31.4%)	15 (32.6%)	1
Pregnancy loss rate, n (%)	34 (32.7%)	36 (32.7%)	1	0 (0.00%)	1 (20.0%)	1	3 (18.8%)	3 (20.0%)	1
Live birth rate, n (%)	76 (26.3%)	72 (25.7%)	0.950	5 (26.3%)	4 (28.6%)	1	13 (25.5%)	12 (26.1%)	1

Numerical variables that follow a normal distribution are reported as mean (standard deviation), numerical variables that do not follow a normal distribution are reported as median [interquartile range], and categorical variables are reported as numbers (percentage).

Continuous variables are analyzed using the Mann-Whitney U test, while categorical variables are analyzed using the chi-square test or Fisher’s test.

* The total count is based on the number of thawing cycles, with some patients having a second thawing cycle.

### Reanalysis of FET cycle clinical outcomes for patients receiving the PPOS regimen

Analysis of FET outcomes in PPOS patients stratified by age revealed no difference in clinical outcomes between the Kuntai group and the non-Kuntai group at different age stages (**Table 6**). Considering that the number of thawing and transplantation cycles is relatively small for patients receiving antagonists and microstimulation, no age stratification analysis was conducted.

**Table 6:** Frozen-thawed embryo transfer clinical outcomes stratified by age in PPOS group.

	<35 year			35–37 year			>37 year		
	Without kuntai (n=129^*^)	With kuntai (n=127^*^)	*p*	Without kuntai (n=56^*^)	With kuntai (n=59^*^)	*p*	Without kuntai (n=136^*^)	With kuntai (n=125^*^)	*p*
Endometrial preparation protocol, n (%)			0.404			0.583			0.185
Artificial cycle	38 (29.5%)	44 (34.6%)		18 (32.1%)	22 (37.3%)		38 (27.9%)	39 (31.2%)	
Downregulated artificial cycle	80 (62.0%)	71 (55.9%)		30 (53.6%)	32 (54.2%)		74 (54.4%)	54 (43.2%)	
Micro stimulation cycle	1 (0.78%)	4 (3.15%)		0 (0.00%)	0 (0.00%)		2 (1.47%)	1 (0.80%)	
Natural cycle	10 (7.75%)	8 (6.3%)		8 (14.3%)	5 (8.47%)		22 (16.2%)	31 (24.8%)	
Endometrial thickness (mm)	9.50 [8.60, 10.9]	9.50 [8.45, 10.8]	0.841	9.47 (1.74)	9.68 (1.93)	0.545	9.35 [7.97, 10.3]	8.70 [7.80, 9.90]	0.067
No. of embryos transferred	1.81 (0.41)	1.71 (0.46)	0.053	1.73 (0.49)	1.63 (0.49)	0.250	1.88 (0.45)	1.78 (0.51)	0.096
Stage of embryos transferred, n (%)			0.084			1.000			0.027
Cleavage	113 (87.6%)	100 (78.7%)		46 (82.1%)	48 (81.4%)	0.617	130 (95.6%)	109 (87.2%)	0.617
Blastocyst	16 (12.4%)	27 (21.3%)		10 (17.9%)	11 (18.6%)	0.730	6 (4.41%)	16 (12.8%)	0.730
Biochemical pregnancy rate, n (%)	83 (64.3%)	76 (59.8%)	0.540	38 (62.5%)	36 (61.0%)	0.568	65 (47.8%)	52 (41.6%)	0.379
Implantation rate, n (%)	94 (40.2%)	85 (39.2%)	0.904	37 (38.9%)	36 (37.5%)	0.955	43 (16.9%)	44 (19.8%)	0.474
Clinical pregnancy rate, n (%)	68 (52.7%)	70 (55.1%)	0.794	30 (54.5%)	31 (52.5%)	0.979	38 (27.9%)	40 (32.0%)	0.562
Pregnancy loss rate, n (%)	10 (17.9%)	12 (24.0%)	0.590	5 (27.8%)	6 (25.0%)	1	14 (46.7%)	18 (50.0%)	0.982
Live birth rate, n (%)	46 (39.3%)	38 (35.5%)	0.653	14 (31.8%)	18 (34.6%)	0.942	16 (12.5%)	18 (14.9%)	0.916

Numerical variables that follow a normal distribution are reported as mean (standard deviation), numerical variables that do not follow a normal distribution are reported as median [interquartile range], and categorical variables are reported as numbers (percentage).

Continuous variables are analyzed using the Mann-Whitney U test, while categorical variables are analyzed using the chi-square test or Fisher’s test.

* The total count is based on the number of thawing cycles, with some patients having a second thawing cycle.

Multivariate logistic regression GEE model analysis was conducted on the clinical pregnancy rate and live birth rate of FET cycles in PPOS patients. The results revealed that there was no difference in the clinical pregnancy rate (OR=1.08, 95% CI 0.77–1.52, p=0.641) or live birth rate (OR=1.03, 95% CI 0.69–1.55, p=0.869) between the group that used Kuntai and the group that did not use Kuntai (**Table 7**).

**Table 7:** Multivariate logistic regression GEE model with odds ratios for frozen-thawed embryo transfer clinical pregnancy and live birth in PPOS group.

	Clinical pregnancy		Live birth	
	Adjusted odds ratio (95% CI)	*p*	Adjusted odds ratio (95% CI)	*p*
Treatment (with kuntai vs. without kuntai)	1.08 (0.77, 1.52)	0.641	1.03 (0.69, 1.55)	0.869
Age (year)				
<35 (reference)				
35-37	1.07 (0.69, 1.68)	0.756	0.90 (0.54, 1.50)	0.678
>37	0.40 (0.28, 0.59)	0.000	0.27 (0.17, 0.43)	0.000
BMI (kg/m^2^)				
<18.5 (reference)				
18.5-24	1.27 (0.64, 2.52)	0.492	1.58 (0.70, 3.56)	0.270
>24	1.53 (0.73, 3.24)	0.266	1.50 (0.61)	0.380
Endometrial preparation protocol				
Artificial cycle (reference)				
Downregulated artificial cycle	1.37 (0.94, 1.98)	0.102	1.02 (0.67, 1.56)	0.920
Micro stimulation cycle	0.44 (0.08, 2.53)	0.355	0.42 (0.05, 3.35)	0.412
Natural cycle	0.58 (0.32, 1.07)	0.081	0.66 (0.31, 1.41)	0.285
Reason				
Male factor (reference)				
other	1.25 (0.81, 1.94)	0.307	1.22 (0.72, 2.09)	0.459
Tubal factor	1.36 (0.87, 2.12)	0.176	1.55 (0.90, 2.67)	0.117
No. of embryos transferred	1.71 (1.07, 2.73)	0.023	2.02 (1.24, 3.30)	0.005
Phase of embryo transferred (blastocyst vs. cleavage embryo)	3.52 (1.89, 6.58)	0.000	2.22 (1.10, 4.48)	0.026

Other infertility factors include ovulation disorders, endometriosis, uterine factors, and so on.

## Discussion

This study analyzed the clinical efficacy of pretreatment with and without Kuntai capsules in DOR patients who underwent IVF/ICSI and FET via the PPOS regimen, antagonist regimen, and microstimulation regimen. The results showed that the use of Kuntai capsules did not significantly improve the number of retrieved eggs in DOR patients or improve clinical outcomes.

As the proportion of DOR increases, how to improve the success rate of DOR patients is increasingly receiving attention from reproductive medicine experts (15–17). This study aimed to compare the clinical efficacy of Kuntai capsule pretreatment for IVF/ICSI in women with different ovulation induction regimens. To our knowledge, this retrospective study of 1474 samples is the largest analysis comparing the use of Kuntai capsule pretreatment with IVF/ICSI in DOR patients. We use the PSM method to control for potential confounding factors in the control group and experimental group studies. The PSM method is useful for observational studies where treatment allocation is nonrandomized and can be seen as a way to seek replication of randomized allocation in routine randomized controlled trials (RCT) (18). Observational studies of ART differ from other studies because each woman has multiple treatment cycles, which can lead to clustering effects. Therefore, the GEE model was used instead of traditional logistic regression for multivariate analysis (19).

There is limited evidence on whether Kuntai pretreatment can improve the outcomes of IVF/ICSI in DOR patients. Most studies have shown that the use of Kuntai can improve the ovarian reserve in DOR patients (9, 11, 20). One RCT showed that the use of Kuntai in DOR patients can increase the number of retrieved eggs (4.2 ± 1.9 VS. 5.1 ± 1.8, p>0.05) and clinical pregnancy rates (38% VS. 20%, p>0.05) (11). One RCT showed that the use of Kuntai in poor ovarian response (POR) patients can improve the number of retrieved eggs (4.54 ± 1.17 VS. 3.71 ± 0.99, p=0.002) and embryo quality (82.61% vs 70.45%, p=0.040) (13). Different from our study design, these two RCTs (1): enrolled smaller patient cohorts (study 1: n=108; study 2: n=70), (2) implemented longer pretreatment periods (three menstrual cycles before IVF/ovulation induction), and (3) exclusively employed fresh embryo transfer protocols. Our study included a large number of patients (n=1474), and Kuntai was used for a short period of time (only during ovulation induction) with a whole embryo freezing strategy, which is why this research results are inconsistent with previous RCT studies. This study did not find that Kuntai pretreatment could increase the number of eggs retrieved from DOR patients or that Kuntai pretreatment could improve clinical outcomes.

Currently, there are no guidelines or consensus recommendations for ovarian stimulation regimens suitable for DOR patients. The PPOS regimen, antagonist regimen, and microsimulation regimen all have the advantages of preventing premature LH surge, preventing ovarian hyperstimulation, and having good efficacy and relatively low cost, making them important ovulation induction regimens for DOR patients (7, 17, 21). Previous studies have not focused on distinguishing between different ovulation induction regimens. Our motivation for conducting this study was to speculate that Kuntai pretreatment can improve outcomes and is related to ovulation induction regimens. However, we did not observe any beneficial therapeutic effects of Kuntai pretreatment in these three ovarian stimulation regimens (live birth, adjust OR 1.03, 95% CI 0.69-1.55, p=0.869). Age is an important factor affecting the number of retrieved eggs and the success rate of patients (22). We speculate that the benefits of Kuntai preprocessing are age related. We conducted a stratified analysis of PPOS patients by age but did not observe any differences in outcomes between the Kuntai pretreatment group and the non-Kuntai pretreatment group (live birth rate, in ppos regimen: <35 year, 39.3% VS. 35.5%, p=0.653; 35–37 year, 31.8% VS. 34.6%, p=0.942; >37 year, 12.5% VS. 14.9%, p=0.916).

This article has some interesting findings. This study observed that in the PPOS regimen group, compared to patients without Kuntai, those Kuntai group patients exhibited a lower number of oocytes retrieved (2.00 [1.00;4.00] VS. 2.00 [1.00;3.00], p<0.001), a similar MII egg rate (85.1% VS. 85.4%, p=0.874) but a higher usable embryo rate (69.2% VS. 74.5%, p=0.012). The Mann-Whitney U test compares the distributions of two groups, not solely their medians. While the medians were identical (2.00), the interquartile ranges (IQRs) differed between groups (without Kuntai group (1.00–4.00) VS. Kuntai group (1.00–3.00)). This suggests that the Kuntai group exhibited a narrower distribution with fewer high outliers (e.g., patients with >3 oocytes). The large sample size (n=596 per group) amplified the detection of subtle distributional differences, leading to a highly significant p-value despite minimal median differences. Although statistically significant, the clinical relevance of this difference is likely limited. Also, the Kuntai group had poorer baseline FSH (8.21 [6.41, 11.1] VS. 9.06 [6.83, 12.3], p<0.001) and lower AMH (0.63 [0.39, 0.91] VS. 0.54 [0.29, 0.81], p<0.001) before PSM than the group without Kuntai. The main reason is that Kuntai pretreatment is more likely used for patients with poorer prognoses, e.g., very low ovarian reserve (9). Although PSM and multiple logistic regression GEE models balance confounding factors, some subtle differences cannot be reflected in the data. So, we speculate the reason for the lower number of retrieved eggs but similar MII egg rate and increased availability of embryos in the Kuntai group may be (1): the large matched cohort increased statistical power to detect even minor deviations in distributions. And such statistical differences may not always equate to clinical importance. (2) patients in the Kuntai group had poorer ovarian reserve, resulting in fewer oocytes retrieved, but this did not compromise oocyte quality. (3) embryologists might have adopted less stringent criteria for embryo transfer and freezing in patients with poorer ovarian reserve to ensure embryo availability. So, the observed statistical significance likely arises from a combination of distributional differences in the data, the large sample size, and proactive selection by clinicians and embryologists, rather than a clinically meaningful improvement in oocyte retrieval.

Therefore, we believe that the results of this study are conservative and that a large prospective cohort study or RCT is urgently needed for more accurate comparisons. This study has another limitation. Owing to the limited application of preimplantation genetic testing at our center, embryo selection is mainly based on morphological grading. Therefore, we cannot rule out confounding effects caused by embryonic aneuploidy.

In summary, this retrospective study supports the hypothesis that pretreatment with Kuntai does not increase the number of retrieved eggs or improve clinical outcomes in DOR patients before IVF/ICSI-FET treatment with PPOS, antagonists, or microstimulation regimens. Owing to the limitations of retrospective studies, this conclusion needs to be confirmed through prospective studies.

## Data Availability

The original contributions presented in the study are included in the article/supplementary material. Further inquiries can be directed to the corresponding author.

## References

[1] Zhu Q Li Y Ma J Ma H Liang X. Potential factors result in diminished ovarian reserve: a comprehensive review. J Ovarian Res. (2023) 16:208. doi: 10.1186/s13048-023-01296-x 37880734 PMC10598941

[2] Cedars MI. Managing poor ovarian response in the patient with diminished ovarian reserve. Fertility sterility. (2022) 117:655–6. doi: 10.1016/j.fertnstert.2022.02.026 35367010

[3] Jiao Z Bukulmez O. Potential roles of experimental reproductive technologies in infertile women with diminished ovarian reserve. J assisted Reprod Genet. (2021) 38:2507–17. doi: 10.1007/s10815-021-02246-6 PMC8581103 34100154

[4] Lin L Chen G Liu Y. Value of estrogen pretreatment in patients with diminished ovarian reserve and elevated FSH on a line antagonist regimen: a retrospective controlled study. J Ovarian Res. (2024) 17:114. doi: 10.1186/s13048-024-01415-2 38802887 PMC11129493

[5] Ata B Capuzzo M Turkgeldi E Yildiz S La Marca A. Progestins for pituitary suppression during ovarian stimulation for ART: a comprehensive and systematic review including meta-analyses. Hum Reprod update. (2021) 27:48–66. doi: 10.1093/humupd/dmaa040 33016316

[6] Massin N. New stimulation regimens: endogenous and exogenous progesterone use to block the LH surge during ovarian stimulation for IVF. Hum Reprod update. (2017) 23:211–20. doi: 10.1093/humupd/dmw047 28062551

[7] Wang M Li L Zhu H Wang R Liu R Zhang H. Comparison of progestin-primed ovarian stimulation regimen and antagonist regimen in women aged 35 years or older with diminished ovarian reserve: A propensity score-matched study. Int J gynaecology obstetrics: Off Organ Int Fed Gynaecology Obstetrics. (2024) 167:162–8. doi: 10.1002/ijgo.v167.1 38619107

[8] Li J Li Y Li M Zhao X Zheng W Zhang J. Analysis of cumulative live birth rate outcomes of three ovarian stimulation protocols in patients after laparoscopic cystectomy of ovarial endometrioma: a retrospective cohort study. Reprod Health. (2023) 20:126. doi: 10.1186/s12978-023-01671-3 37644567 PMC10464272

[9] Zhang X Zhang L Xiong L Liu X Zhang J Yu F. Kuntai capsule for the treatment of diminished ovarian reserve: A systematic review and meta-analysis of randomized controlled trials. J ethnopharmacology. (2024) 329:118167. doi: 10.1016/j.jep.2024.118167 38593964

[10] Jiang GL Wan P An XQ Yu WT Wang P Zhou XM. Efficacy of supplemented Er-xian decoction combined with acupoint application for poor ovarian response. J Physiol pharmacology: an Off J Polish Physiol Soc. (2020) 71. doi: 10.26402/jpp.2020.2.09 32776907

[11] Gao H Xia T Ma R Zhao Z Wang B Song X. Heyan Kuntai capsule versus dehydroepiandrosterone in treating Chinese patients with infertility caused by diminished ovarian reserve: a multicenter, randomized controlled trial. J traditional Chin Med = Chung i tsa chih ying wen pan. (2017) 37:530–7. 32188212

[12] Du X Xu L Wang L Heng M Bu H Hao Y. Comparison of the effect and safety of Kuntai capsule and hormone replacement therapy in patients with perimenopausal syndrome: a systematic review and Meta-analysis. J traditional Chin Med = Chung i tsa chih ying wen pan. (2017) 37:279–85. 31682369

[13] Lian F Jiang XY. Effect of kuntai capsule on the number of retrieved oocytes, high-quality oocytes and embryos in *in vitro* fertilization of poor ovarian response patients. Zhongguo Zhong xi yi jie he za zhi Zhongguo Zhongxiyi jiehe zazhi = Chin J integrated traditional Western medicine. (2014) 34:917–21. 25223172

[14] Xia L Tian L Zhang S Huang J Wu Q. Hormonal replacement treatment for frozen-thawed embryo transfer with or without gnRH agonist pretreatment: A retrospective cohort study stratified by times of embryo implantation failures. Front endocrinology. (2022) 13:803471. doi: 10.3389/fendo.2022.803471 PMC8850772 35185793

[15] Lin G Li X Jin Yie SL Xu L. Clinical evidence of coenzyme Q10 pretreatment for women with diminished ovarian reserve undergoing IVF/ICSI: a systematic review and meta-analysis. Ann medicine. (2024) 56:2389469. doi: 10.1080/07853890.2024.2389469 PMC11321116 39129455

[16] Zhang J Jia H Diao F Ma X Liu J Cui Y. Efficacy of dehydroepiandrosterone priming in women with poor ovarian response undergoing IVF/ICSI: a meta-analysis. Front endocrinology. (2023) 14:1156280. doi: 10.3389/fendo.2023.1156280 PMC10288189 37361534

[17] Lin G Zhong X Li S Liu X Xu L. The clinical value of progestin-primed ovarian stimulation protocol for women with diminished ovarian reserve undergoing IVF/ICSI: a systematic review and meta-analysis. Front endocrinology. (2023) 14:1232935. doi: 10.3389/fendo.2023.1232935 PMC10476097 37670890

[18] Whittaker W Anselmi L Kristensen SR Lau YS Bailey S Bower P. Associations between extending access to primary care and emergency department visits: A difference-in-differences analysis. PloS medicine. (2016) 13:e1002113. doi: 10.1371/journal.pmed.1002113 27598248 PMC5012704

[19] Xu Z Fine JP Song W Yan J. On GEE for mean-variance-correlation models: variance estimation and model selection. Stat Med. (2025) 44:e10271. doi: 10.1002/sim.v44.1-2 39665136

[20] Lin XM Chen M Wang QL Ye XM Chen HF. Clinical observation of Kuntai capsule combined with Fenmotong in treatment of decline of ovarian reserve function. World J Clin cases. (2021) 9:8349–57. doi: 10.12998/wjcc.v9.i28.8349 PMC8554447 34754844

[21] Chen K Zhang C Chen L Zhao Y Li H. Reproductive outcomes of dual trigger therapy with GnRH agonist and hCG versus hCG trigger in women with diminished ovarian reserve: a retrospective study. Reprod Biol endocrinology: RB&E. (2024) 22:35. doi: 10.1186/s12958-024-01211-z 38566172 PMC10985881

[22] Shingshetty L Cameron NJ McLernon DJ Bhattacharya S. Predictors of success after *in vitro* fertilization. Fertility sterility. (2024) 121:742–51. doi: 10.1016/j.fertnstert.2024.03.003 38492930

